# Safety and Efficacy of Vaccination Against Influenza in Patients With Rheumatoid Arthritis 

**DOI:** 10.1080/17402520600589613

**Published:** 2006

**Authors:** Ori Elkayam

**Affiliations:** Departments of Internal Medicine and Rheumatology Tel Aviv Medical Center and the “Sackler” School of Medicine University of Tel Aviv Israel

## Abstract

Vaccination against influenza is currently recommended for patients with rheumatoid arthritis (RA). The safety and efficacy of vaccination in patients suffering from rheumatic diseases is still a matter of debate. This review summarizes the studies performed on the safety and immunogenicity of influenza vaccination in patients with RA as well as the rheumatic complications of the vaccine in otherwise healthy persons. Several trials have shown that the vaccine induces an adequate humoral response and does not induce clinical exacerbation of RA. Rheumatic complications (mainly vasculitis) following influenza vaccination in the general population are scarce.


*Clinical & Developmental Immunology*, June–December 2006; 13(2–4): 349–351

Taylor & Francis

Taylor & Francis Group

### Safety and efficacy of vaccination against influenza in patients with rheumatoid arthritis

ORI ELKAYAM

*Departments of Internal Medicine and Rheumatology, Tel Aviv Medical Center and the "Sackler" School of Medicine, University of Tel Aviv, Israel*

**Abstract**
Vaccination against influenza is currently recommended for patients with rheumatoid arthritis (RA). The safety and efficacy of vaccination in patients suffering from rheumatic diseases is still a matter of debate. This review summarizes the studies performed on the safety and immunogenicity of influenza vaccination in patients with RA as well as the rheumatic complications of the vaccine in otherwise healthy persons. Several trials have shown that the vaccine induces an adequate humoral response and does not induce clinical exacerbation of RA. Rheumatic complications (mainly vasculitis) following influenza vaccination in the general population are scarce.

**Keywords:** *Rheumatoid, influenza, vaccine, efficacy, safety*

Yearly influenza vaccination is currently recommended for patients with rheumatoid arthritis (RA) (Center for Disease Control and Prevention 2002). Despite this specific recommendation, the uptake of influenza vaccination in patients with RA is low (Bridges et al. 2003). The principal reason seems not to be a lack of awareness of the need of vaccination by the patient, but the fact that they have not been offered it (Bridges et al. 2003).

This reluctance to vaccinate RA patients is based on sporadic case reports on the onset or exacerbation of RA following vaccination with influenza, tetanus, hepatitis and other vaccines (Older et al. 1999, Maillefert et al. 1999). However, the eventual capacity of influenza vaccination to induce a significant clinical flare of RA is still debated. One study reported post vaccination flare in 6 out of 17 patients (Herron et al. 1979) while no flares were reported in further studies (Arnold and Hochberg 1989, Turner Strokesluenza et al. 1988, Chalmers et al. 1994).

On the other hand, there is uncertainty concerning the immunogenicity of vaccination in immunocompromised patients such as RA patients.

The present report summarizes the current knowledge on the efficacy and safety of vaccination against influenza in RA patients.

### Trials on the effect of vaccination against influenza in RA patients

### *Adult RA patients*

The first study on the safety and efficacy of influenza vaccination was reported in 1979 (Herron et al. 1979). The subjects participating in this study were 62 patients with systemic lupus erythematosus (SLE), RA, degenerative joint disease and 32 healthy volunteers. Flare-ups of rheumatic disease following immunization were infrequent and usually minor. Seroconversion to A/New Jersey/76 developed in 62–87% of all individuals and to A/Victoria/75 in 62–69%. Administration of this vaccine induced an adequate antibody response in both healthy and rheumatic patients (Herron et al. 1979).

Chalmers et al. have addressed the issue of immunogenicity and safety in 126 RA patients

Correspondence: O. Elkayam, Department of Rheumatology, Tel Aviv Medical Center, 6 Weizman Street Tel Aviv, Tel Aviv 64239, Israel. Fax: 972 36974577. E-mail: oribe14@netvision.net.il

ISSN 1740-2522 print/ISSN 1740-2530 online © 2006 Taylor & Francis
DOI: 10.1080/17402520600589613

stratified in three groups: (1) those with a history of vaccination with influenza vaccine within 24 months who were receiving usual therapy for RA (2) those receiving usual therapy without prior vaccine and (3) those receiving immunosuppressive medication or prednisone $>7.5$ mg/day. Within each group, patients were randomized to receive vaccine or placebo. During 1-month follow-up period, adverse reactions occurred with equal frequency among patients with RA and healthy controls. Similar significant increases in titers to the vaccine were seen in all groups of patients with arthritis and in the controls (Chalmers et al. 1994).

Our group has studied the effect of influenza vaccination in 82 RA patients and 30 healthy controls using a split-virion inactivated vaccine containing 15 mcg hemagglutinin (HA)/dose of each of B/Hong Kong/330 (hk), A/Panama/2007/99 (pan) and A/New Caledonian /20/90 (nc) (Fomin et al. 2006). Six weeks after vaccination, a significant increase in geometric mean titer of hemaglutination inhibition antibodies for each antigen was observed in both groups, the increase being significantly higher in the healthy group for hk ($p = 0.004$). The percentage of responders was lower in RA patients in comparison with healthy controls achieving statistical significance for hk. Parameters of disease activity such as number of tender and swollen joints, morning stiffness, evaluation of pain, health assessment questionnaire, ESR and CRP remained unchanged. Our study clearly showed that influenza virus vaccine generated a good humoral response in RA patients, although lower than in healthy controls (Fomin et al. 2006).

### *Children with chronic arthritis*

Malleson et al. have reported their experience with influenza vaccination using an inactivated split virus vaccine prepared for the 1991/1992 season in a prospective open study of 34 children with chronic arthritis and 11 healthy control children. Tenderness and/or redness at the injection site occurred equally in patients and controls. Malaise/nausea occurred in 12 patients and three controls but patients had more symptomatic days than controls. No significant worsening of indices of disease activity such as morning stiffness, pain, joint count and ESR were observed. At least 95% of patients developed presumably protective levels of antibodies. Preimmunization titers, seroresponse rates and final titers were the same between patients and controls (Malleson et al. 1993).

A subsequent report published in 2001 has confirmed the safety and efficacy of influenza vaccination in 70 children with chronic rheumatic diseases (Juvenile RA 49, SLE 11, other 10) aged 4–17 years and five healthy siblings who received a split type influenza vaccine for the 1999–2000 winter season. Five out of 70 patients reported local (three patients) or systemic (two patients) reactions and 1/5 siblings local reaction. Nine more patients reported mild upper respiratory tract symptoms 1–4 weeks post-vaccination. No patient was found to fulfill criteria for deterioration or flare of the underlying disease.

At completion of vaccination 97% of patients developed protective HI titers to A/Beijing, 100% to A/Sydney and 80% to B/Beijing (Kanakoudi-Tsakalidou et al. 2001).

### The effect of immunosuppressive drugs on the efficacy of influenza vaccination in RA patients

Various studies have addressed the effect of glucocorticoids on the immunogenicity of the vaccine (Malleson et al. 1993, Chalmers et al. 1994, Kanakoudi-Tsakalidou et al. 2001). They all showed that glucocorticoids did not significantly affect the development of protective antibodies (Malleson et al. 1993, Chalmers et al. 1994, Kanakoudi-Tsakalidou et al. 2001). The same was demonstrated for gold, imuran and methotrexate (Chalmers et al. 1994, Fomin et al. 2006).

Our group has found that the use of infliximab did not seem to influence the humoral response to influenza vaccine.

Thus, it seems that the use of immunosuppressive drugs, at the dosage used to treat patients with RA, does not affect the efficacy of vaccination against influenza.

### Rheumatic complications of influenza vaccination

Vasculitis is by far the most common rheumatic complication following influenza vaccination. Although this association is far from being new (Wharton and Pietroni 1974), it is of importance in that small, medium or large vessels can apparently be involved (Ghose et al. 1976, Blumberg et al. 1980, Kelsall et al. 1997, Perez et al. 2000, Finsterer et al. 2001, Iyngkaran et al. 2003). Blumberg has described two patients who experienced small-vessel vasculitic syndromes after influenza immunization (Blumberg et al. 1980). Their illnesses were characterized by fever, arthralgias and myalgias; uveitis and optic neuritis occurred in one patient, while the other had palpable purpura, which histologically was proved to be a cutaneous necrotizing venulitis. Kelsall et al. (1997) reported a case of microscopic polyangiitis that developed after influenza vaccination, with increased titers of anti-influenza A antibody in synovial fluid. Their review of the literature found 16 cases of vasculitis that occurred after IV, which were reclassified according to the Chapel Hill diagnostic criteria as follows: microscopic polyangiitis, Churg-Strauss syndrome, cutaneous leukocytoclastic vasculitis, and

Henoch-Schönlein purpura. Giant cell arteritis occurring shortly after influenza vaccination has been described in three cases (Ghose et al. 1976, Perez et al. 2000, Finsterer et al. 2001) further expanding the spectrum of vasculitis induced by vaccination. A single case of rheumatoid vasculitis triggered by influenza vaccination in a patient suffering from RA has been reported (Iyngkaran et al. 2003).

Sporadic cases of arthritis, including polyarthritis (Thurairajan et al. 1997) and reactive arthritis (Biasi et al. 1994) complete the repertoire of rheumatic complications of influenza vaccination.

### Conclusion

The effect of vaccination against influenza in RA has been investigated in several trials. They all conclude that the vaccine is safe, does not induce an exacerbation of the disease and achieves an immunogenic response similar to the one observed in healthy subjects, although a trend toward a lower response has been reported in some of the studies. The effect of glucocorticoids and disease-modifying drugs, including infliximab, on the humoral response seems to be negligible. Vaccination against influenza has been associated with sporadic cases of vasculitis and arthritis. Influenza vaccination may have the capability to induce rheumatic complications in predisposed patients. However, the scarcity of the reports in comparison with the huge number of recipients of the flu vaccine all over the years is reassuring.

### References

Arnold CM, Hochberg MC. 1989. Development of an immunization program for patients with rheumatoid arthritis. Arthritis Care Res 2:162–164.

Biasi D, Carletto A, Caramaschi P, Tonoli M, Bambara LM. 1994. A case of reactive arthritis after influenza vaccination. Clin Rheumatol 13(4):645.

Blumberg S, Bienfang D, Kantrowitz FG. 1980. A possible association between influenza vaccination and small-vessel vasculitis. Arch Intern Med 140(6):847–848.

Bridges MJ, Coady D, Kelly CA, Hamilton J, Heycock C. 2003. Factors influencing uptake of influenza vaccination in patients with rheumatoid arthritis. Ann Rheum Dis 62(7):685.

Center for Disease Control and Prevention: Prevention and control of influenza, recommendation of the Advisory Committee on Immunization Practice. 2002. Morb Mortal Wkly Rep

Chalmers A, Scheifele D, Patterson C, Williams D, Weber J, Shuckett R, Teufel A. 1994. Immunization of patients with rheumatoid arthritis against influenza: A study of vaccine safety and immunogenicity. J Rheumatol 21(7):1203–1206.

Finsterer J, Artner C, Kladosek A, Kalchmayr R, Redtenbacher S. 2001. Cavernous sinus syndrome due to vaccination-induced giant cell arteritis. Arch Intern Med 161(7):1008–1009.

Fomin I, Caspi D, Shalev Y, Levy V, Mendelson E, Paran D, Levartovsky D, Litinsky I, Kaufman I, Wigler I, Elkayam O. 2006. Vaccination against influenza in rheumatoid arthritis: The effect of disease modifying drugs, including TNF α blockers. Ann Rheum Dis 65(2):191–194.

Ghose MK, Shensa S, Lerner PI. 1976. Arteritis of the aged (giant cell arthritis) and fever of unexplained origin. Am J Med 60:429–436.

Herron A, Dettleff G, Hixon B, et al. 1979. Influenza vaccination in patients with rheumatic diseases. JAMA 242:53–56.

Iyngkaran P, Limaye V, Hill C, Henderson D, Pile KD, Rischmueller M. 2003. Rheumatoid vasculitis following influenza vaccination. Rheumatology 42:907–909.

Kanakoudi-Tsakalidou F, Trachana M, Pratsidou-Gertsi P, Tsitsami E, Kyriazopoulou-Dalaina V. 2001. Influenza vaccination in children with chronic rheumatic diseases and long-term immunosuppressive therapy. Clin Exp Rheumatol 19(5):589–594.

Kelsall JT, Chalmers A, Sherlock CH, Tron VA, Kelsall AC. 1997. Microscopic polyangiitis after influenza vaccination. J Rheumatol 24(6):1198–1202.

Maillefert JF, Sibilia J, Toussirot E, Vignon E, Eschard JP, Lorcerie B, Juvin R, Parchin-Geneste N, Piroth C, Wendling D, Kuntz JL, Tavernier C, Gaudin P. 1999. Rheumatic disorders developed after hepatitis B vaccination. Rheumatology 38:978–983.

Malleson PN, Tekano JL, Scheifele DW, Weber JM. 1993. Influenza immunization in children with chronic arthritis: A prospective study. J Rheumatol 20(10):1769–1773.

Older SA, Battafarano DF, Enzenauer RJ, et al. 1999. Can immunization precipitate connective tissue disease? Report of five cases and review of the literature. Semin Arthritis Rheum 29:131–139.

Perez C, Loza E, Tinture T. 2000. Giant cell arthritis after influenza vaccination [letter]. Arch Intern Med 160:2677.

Thurairajan G, Hope-Ross MW, Situnayake RD, Murray PI. 1997. Polyarthropathy, orbital myositis and posterior scleritis: An unusual adverse reaction to influenza vaccine. Br J Rheumatol 36(1):120–123.

Turner Strokesluenza L, Cambridge G, Corcoran T, Oxford JS, Snaith ML. 1988. *In vitro* response to influenza immunization by peripheral blood mononuclear cells from patients with systemic lupus erythematosus and other autoimmune diseases. Ann Rheum Dis 47:532–535.

Wharton CF, Pietroni R. 1974. Polyarteritis after influenza vaccination. BMJ :331–332.

